# F88. MANIPULATION OF THE GUT MICROBIOTA WITH A PREBIOTIC IN SCHIZOPHRENIA: A DOUBLE-BLINDED RANDOMIZED PLACEBO-CONTROLLED CROSS-OVER STUDY

**DOI:** 10.1093/schbul/sby017.619

**Published:** 2018-04-01

**Authors:** Amy Kao, Phil Burnet, Belinda Lennox

**Affiliations:** University of Oxford

## Abstract

**Background:**

Neurocognitive impairment is increasingly recognized as a fundamental symptomatology in schizophrenia with more than 80% of patients exhibiting significant deficits, even at first episode of illness. Current pharmacotherapies do not alleviate cognitive symptoms, and often cause severe metabolic dysfunction and weight gain that readily become major health concerns. Identifying adjunctive interventions that improve cognition without disrupting the therapeutic actions of anti-psychotic medications, and can mitigate the metabolic side-effects of these drugs, would be highly beneficial to patients and improve prognosis. We have recently demonstrated that the manipulation of the rat gut microbiota with a prebiotic (dietary fibre that grows beneficial enteric bacteria) improves cognitive flexibility and prevents olanzapine-mediated weight gain. We therefore aim to explore whether these actions of the prebiotic translate to medicated stable patients with schizophrenia.

**Methods:**

A total of 40 patients with psychosis aged 18–65 will be enrolled in a 24-week maltodextrin-controlled cross-over experimental medicine study. Participants will receive either a 12-week treatment with a prebiotic (active compound) followed by a 12-week maltodextrin supplement (placebo), or in the reverse order. The order of supplements that participants receive is randomized. The primary outcome is to examine the influence of prebiotic supplementation on neurocognitive functioning, which is measured using a tablet-based neuropsychometric test battery. We will also examine the impact on clinical metabolic measures such as total weight and visceral adiposity. The concentration of immune-related serum proteins as well as neuroendocrine hormones will be evaluated. All measurements will take place at baseline, at 12-week cross-over, and at the end of the 24-week study. A within-subjects repeated measures analysis will be performed, and co-variates (gender, weight, medication) identified. This trial is registered with ClinicalTrials.gov, identifier number NCT03153046.

**Results:**

We have currently screened 36 patients, of whom 30 were eligible for the study (67% male). At baseline, the average age of all recruited was 36.41 ± 11.42. The overall cognitive score was -2.03 ± 0.53 where the subtests included verbal memory (29.09 ± 8.75), digit sequencing (15.41 ± 2.77), token motor (55.74 ± 24.14), semantic fluency (8.83 ± 3.46), letter fluency (11.39 ± 3.65), symbol coding (35.61 ± 9.22) and tower of London (15.1 ± 4.06). There was no difference in overall cognitive between male (14.29 ± 2.46) and female (17.22 ± 2.30) patients. However, long-term associative learning as measured by the digit sequencing subtest appeared to show a significant difference between male (14.29 ± 2.46) and female (17.22 ± 2.33; p=0.008) participants. No significant differences between clinical metabolic measures were observed in baseline BMI (32.25 ± 6.79) and abdominal obesity as measured by hip-to-waist ratio (0.94 ± 0.11). The serum concentrations of immune and endocrine markers will also be presented.

**Discussion:**

This investigation, to our knowledge, is the first clinical study to provide medicated schizophrenia patients with a prebiotic, as a potential means of improving cognition and managing secondary metabolic dysfunction. Although a potential link between commensal enteric bacteria and schizophrenia have been suggested, earlier work with probiotics (live cultures) did not support this association. However, since the current study uses a prebiotic that proliferates multiple species of gut bacteria, it will provide more robust data that will support, or refute, the validity of manipulating the gut microbiome in the treatment of schizophrenia.

